# Tumor characteristics of dissociated response to immune checkpoint inhibition in advanced melanoma

**DOI:** 10.1007/s00262-023-03581-6

**Published:** 2024-01-27

**Authors:** J. M. Versluis, E. P. Hoefsmit, H. Shehwana, P. Dimitriadis, J. Sanders, A. Broeks, C. U. Blank

**Affiliations:** 1 Department of Medical Oncology, Netherlands Cancer Institute, Amsterdam, The Netherlands; 2 Division of Molecular Oncology & Immunology, Netherlands Cancer Institute, Amsterdam, The Netherlands; 3 Department of Pathology, Netherlands Cancer Institute, Amsterdam, The Netherlands; 4 Core Facility Molecular Pathology and Biobanking, Netherlands Cancer Institute, Amsterdam, The Netherlands; 5 Department of Medical Oncology, Leiden University Medical Center, Leiden, The Netherlands

**Keywords:** Melanoma, Immune checkpoint inhibition, Dissociated response, Acquired resistance

## Abstract

**Introduction:**

Immune checkpoint inhibition (ICI) has improved patients’ outcomes in advanced melanoma, often resulting in durable response. However, not all patients have durable responses and the patients with dissociated response are a valuable subgroup to identify mechanisms of ICI resistance.

**Methods:**

Stage IV melanoma patients treated with ICI and dissociated response were retrospectively screened for available samples containing sufficient tumor at least at two time-points. Included were one patient with metachronous regressive and progressive lesions at the same site, two patients with regressive and novel lesion at different sites, and three patients with regressive and progressive lesions at different sites. In addition, four patients with acquired resistant tumor samples without a matched second sample were included.

**Results:**

In the majority of patients, the progressive tumor lesion contained higher CD8^+^ T cell counts/mm^2^ and interferon-gamma (IFN*γ*) signature level, but similar tumor PD-L1 expression. The tumor mutational burden levels were in 2 out 3 lesions higher compared to the corresponding regressive tumors lesion.

In the acquired tumor lesions, high CD8^+^/mm^2^ and relatively high IFNγ signature levels were observed. In one patient in both the B2M and PTEN gene a stop gaining mutation and in another patient a pathogenic POLE mutation were found.

**Conclusion:**

Intrapatient comparison of progressive versus regressive lesions indicates no defect in tumor T cell infiltration, and in general no tumor immune exclusion were observed.

**Supplementary Information:**

The online version contains supplementary material available at 10.1007/s00262-023-03581-6.

## Introduction

Immune checkpoint inhibition (ICI) has shown improvement in overall survival (OS) in a broad range of advanced malignancies and has become a standard treatment option, amongst others, for stage IV melanoma patients [1–7]. In a subset of patients treated with ICI durable responses are seen, which can persist after (early) treatment discontinuation [8, 9] and result in a plateau of 36% for anti-CTLA-4 plus anti-PD-1 combination and 8–29% for anti-PD-1 monotherapy [2].

There are, however, also dissociated response patterns [10]. For instance, mixed response, with some tumor lesions regressing while other persist or increase in size, and acquired resistance, when a tumor lesion initially responds or stays stable for long periods of time, but eventually progression is observed [10, 11]. These heterogeneous clinical patterns of response can be both spatial, with different responses in different tumor lesions as in mixed response, and temporal, with different responses over time [11]. There are different resistant mechanisms to ICI proposed [12]. Innate resistance is defined as initial non-responding tumor lesions to ICI. If a tumor is recognized by the immune system, but it is also capable of adapting and thereby escaping the immune attack, it is termed acquired resistance. Since tumor lesions are constantly evolving, this could either result in innate resistance, mixed response or acquired resistance [12].

The biological mechanisms underlying dissociated response are still being explored. Resistance to ICI can manifest at different times, but in many cases similar or overlapping mechanisms contribute to the immune escape from tumor cells [12]. Acquired resistance can be the result of, e.g., changes in the tumor neoantigen presentation machinery. Due to a selection of non-responsive clones or to mutations resulting in loss of neoantigen, tumor antigen presentation is downregulated, causing a lack of T cell recognition [11, 13]. Alterations in genes encoding for components needed for antigen processing and/or presentation can lead to ICI resistance as well [11]. Other factors, such as an immune suppressive tumor microenvironment and DNA mismatch repair deficiencies, can play a role in resistance mechanisms as well [14]. Understanding of these mechanisms by comparison of paired biopsy samples may guide rational design of salvage therapies or preventive strategies.

In this study, we aimed to assess histopathological, DNA and RNA characteristics of paired biopsies of regressive and progressive tumor lesions, in an effort to gain insight in mechanisms of dissociated response to ICI in a cohort of stage IV melanoma patients.

## Methods

### Patient and material selection

Patients with stage IV melanoma treated in the Netherlands Cancer Institute between 2010 till May 2019 with dissociated response patterns (defined as both regressive and progressive tumor lesions present on CT evaluation scans) to ICI treatment were screened for availability of tumor material. ICI treatment consisted of either pembrolizumab (anti-PD-1 mAb) 2 mg/kg or in a fixed dose of 150–200 mg intravenously every three weeks, nivolumab (anti-PD-1 mAb) 3 mg/kg or in a fixed dose of 240 mg intravenously every three weeks, ipilimumab (anti-CTLA-4mAb) 3 mg/kg every three weeks for a maximum of four cycles, or ipilimumab 1 mg/kg plus nivolumab 1 mg/kg for four cycles followed by maintenance nivolumab 240 mg every three weeks.

Patients with dissociated response, who did not opt out for use of remaining tumor material for research purpose or had given informed consent for performing additional biopsies for research purposes, were screened for availability of tumor samples. When both a regressive baseline tumor sample (taken within 3 months before start treatment) and a tumor sample of a progressive lesion with sufficient tumor cells (at least 30% tumor cells of HE stained frozen section) were present, patients were included.

Clinical characteristics and RECIST response were collected from patient records. This study was conducted in accordance with the Declaration of Helsinki after approval by the local institutional review board.

### Immunohistochemistry

The formalin-fixed, paraffin-embedded (FFPE) samples were stained for both PD-L1 and CD8. Immunohistochemistry of the FFPE tumor samples was performed on a BenchMark Ultra autostainer (Ventana Medical Systems). Briefly, paraffin sections were cut at 3 µm, heated at 75 °C for 28 min and deparaffinized in the instrument with EZ prep solution (Ventana Medical Systems). Heat-induced antigen retrieval was carried out using Cell Conditioning 1 (CC1, Ventana Medical Systems) for 32 min at 95 °C (CD8) or 48 min at 95 °C (PD-L1). CD8 was detected using clone C8/144B (1/100 dilution, 32 min at 37 °C, Agilent/DAKO) and PD-L1 was detected using clone 22C3 (1/40 dilution, 1 h at RT, Agilent/DAKO). Bound antibody was detected using the OptiView DAB Detection Kit (Ventana Medical Systems). Slides were counterstained with Hematoxylin and Bluing Reagent (Ventana Medical Systems). A Pannoramic® 1000 scanner from 3DHISTECH was used to scan the slides at a 40 × magnification. The stained FFPE slides were scored by a blinded pathologist using Slidescore (www.slidescore.com). Of each biopsy, five representative areas of 0.2mm^2^ were selected to assess the number of CD8^+^ cells/mm^2^.

### RNA and DNA sequencing

RNA and DNA were simultaneously isolated from FFPE sections (10 µm) with the AllPrep DNA/RNA FFPE kit (Qiagen, 80,234), according to manufacturers’ protocol, using the QIAcube. Non-tumor DNA to determine mutation load, was isolated from PBMCs using the AllPrep DNA/RNA FF kit (Qiagen, 80,224), and when no PBMCs were available, from whole blood samples, using the MagNa Pure Compact Nucleic Acid Isolation Kit.

Transcriptome and whole-exome sequencing were performed by CeGaT GmbH (Tübingen, Germany). Transcriptome libraries were generated using the KAPA RNA HyperPrep with RiboErase (HMR) & SMART-Seq stranded total RNA (Takara). Exome libraries were generated using the Twist Human Core Exome Plus (Twist Bioscience). These libraries were sequenced with 2 × 100 base pair reads on a NovaSeq 600 system according to manufacturer’s protocols, with a sequence quality value of > 93% for transcriptome and > 90% for exome libraries.

Data were analyzed in the CeGaT analysis pipeline. Briefly, demultiplexing of the sequencing reads was performed with Illumina bcl2fastq (2.20) and adapters trimmed with Skewer (v0.2.2) [15]. The quality of FASTQ files was analyzed with FastQC (v0.11.5-cegat) [16].

FASTQ files with DNA sequencing data were aligned to the human reference genome (GRCh38) using Burrows-Wheeler Aligner [17], duplicate reads were marked by Picard MarkDuplicates. Using GATK BaseRecalibrator base quality scores were recalibrated and single nucleotide variants were called using GATK MuTect2 [18].

To evaluate correct normal-tumor pairing and any underlying contamination in the tumor samples, GATK calculate contamination and BAMix were used. The tumor mutational burden was calculated by summarizing the total number of non-synonymous, somatic mutations per sample with minimal variant allele frequency of 0.05 (5%). FATHMM prediction was used to predict functional consequences of non-coding and coding sequence variation [19].

RNA sequencing data were mapped with STAR (v2.7.3a) [20] to human reference genome using default settings. The read counts were computed with HTseq-count (v0.12.4) [21] and were analyzed with DESeq2 (v1.38.2) [22]. For the downstream analyses, data were analyzed using R (v4.2.2). Centering of normalized gene expression was performed by subtracting the row means and scaling by dividing the columns by the standard deviation. The Danaher immune cell [23], interferon-gamma (IFNγ) [24], micro-environment cell population (MCP counter) [25] gene expression signatures were analyzed and expressed in normalized z-scores.

### Statistical analysis

Descriptive statistics were performed on patient and tumor characteristics using IBM SPSS Statistics, version 27. Overviews of RNA gene expressions levels were visualized with the tidyverse and reshape2 library in R.

## Results

### Patient characteristics

**Fig. 1:**
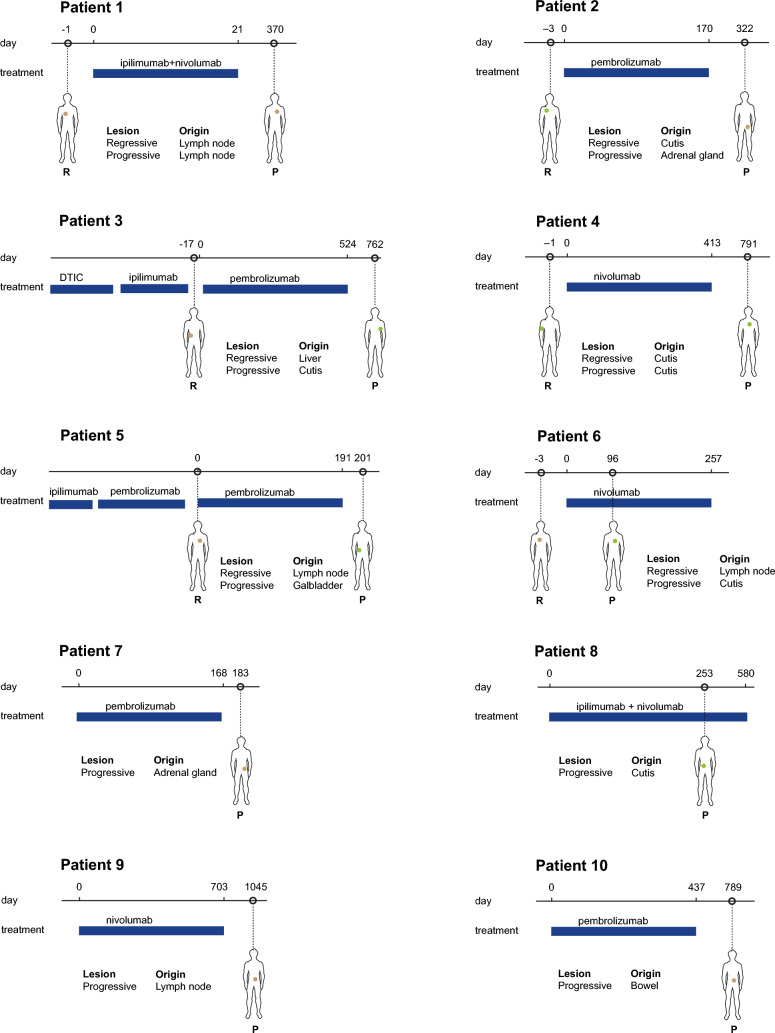
Overview of patients and tumor lesions. Layout inspired in Fig. 1 of Liu et al. [26]

Ten patients were identified with available tumor samples at at least two time-points, with sufficient tumor material to send for sequencing. Of these patients, one patient had metachronous regressive and progressive tumor lesions of the same site (patient 1), two patients had metachronous regressive and novel tumor lesions at another site (patients 2 and 3), and three patients had metachronous regressive and progressive tumor lesions at different sites (patients 4–6) (Fig. 1). In addition, four patients had acquired resistant lesions without a comparative tumor lesion, as due to removal of the other tumor lesion for diagnostic purposes there was no known response of that specific lesion (patients 7–10).

These ten patients were in majority male (80%), had cutaneous melanoma (90%) and were treatment-naïve (80%) (Table 1). Most patients had either BRAFV600E/K (40%) or NRAS (30%) mutation-positive melanoma, and half of the patients received pembrolizumab. All seven patients of whom performance status was recorded at baseline, had a WHO performance status of 0. Details of treatment per patient are depicted in Suppl. Table 1.

**Table 1:** Clinical characteristics of patients included

	Patients (*n* = 10)
Sex, male (*n*, %)	8 (80%)
Age at start ICI (median, range)	68 (35–78)
*Primary melanoma*	
Cutaneous	9 (90%)
Unknown primary	1 (10%)
*Mutational status*	
BRAF V600E/K	4 (40%)
NRAS	3 (30%)
BRAF V600E/K, NRAS + KIT wildtype	3 (30%)
Prior systemic treatment*	2 (20%)
Prior DTIC	1 (10%)
Prior ICI	2 (20%)
*AJCC 8th edition at start ICI*	
M1b	4 (40%)
M1c	4 (40%)
M1d	2 (20%)
LDH < ULN at start ICI	10 (100%)
*ICI regimen*	
Pembrolizumab	5 (50%)
Nivolumab monotherapy	3 (30%)
Ipilimumab + nivolumab	2 (20)
Number of cycles ICI (median, range)	21 (1–53)
*Best overall response to ICI*	
Complete response	1 (10%)
Partial response	6 (60%)
Progressive disease	2 (20%)
Mixed response	1 (10%)

*ICI* immune checkpoint inhibitor, in table this refers to the ICI line on which dissociated response occurred, *IQR* interquartile range, *ULN* upper limit of normal

*One patient had two prior lines of treatment: DTIC and ipilimumab monotherapy

### Characteristics of paired tumor lesions

In five out of six patients with paired tumor lesions, RNA sequencing data were available, while DNA sequencing data were available only in three patients, both due to insufficient material.

In patient 1 (metachronous progressive and regressive lesion), a higher number of CD8^+^ T cells was counted in the progressive lesion (+528 CD8^+^ cells/mm^2^). This was in contrast to both the Danaher and MCP counter immune cell subsets, which demonstrated lower T cell gene expression in the progressive lesion, ranging from −2.83 to −0.97 (Suppl. Fig. 1). In line with the progression, a lower IFN*γ* score (the average of the genes of the IFN*γ* signature, −1.937) was detected in the progressive lesion, while the PD-L1 expression on tumor cells was higher in the progressive lesion (10–50% vs. 1–10%) (Table 2).

**Table 2:** Overview of CD8, PD-L1, IFN*γ* and TMB levels of samples

Patient	Regressive lesion	CD8^+^ cells/mm^2^	PD-L1 tumor cells	IFN*γ* score	TMB level	Progressive lesion	CD8^+^ cells/mm^2^	PD-L1 tumor cells	IFN*γ* score	TMB level
1	Lymph node	314	1–10%	0.641	–	Lymph node	842	10–50%	−1.296	−
2	Cutis	1742	10–50%	0.046	742	Adrenal gland	1629	10–50%	0.534	482
3	Liver	225	< 1%	−2.164	−	Cutis	521	< 1%	−0.231	−
4	Cutis	–	–	0.148	717	Cutis	1094	10–50%	0.877	4588
5	Lymph node	172	1–10%	−1.155	–	Gall bladder	250	< 1%	1.219	−
6	Lymph node	–	–	–	169	Cutis	–	–	−0.388	894
7	–	–	–	–	–	Adrenal gland	762	1–10%	−0.015	741
8	–	–	–	–	–	Cutis	880	10–50%	1.108	553
9	–	–	–	–	–	Lymph node	1161	1–10%	−0.177	327
10	–	–	–	–	–	Bowel	1042	1–10%	0.030	1221

*IFNγ* interferon-gamma, *TMB* tumor mutational burden

Unlike for patient 1, both patients 2 and 3 had higher IFNγ scores (+ 0.488 and + 1.933) in their novel progressive versus the regressive lesion. Again, the CD8 T cell infiltration was similar or higher in the progressive lesion (1742 and 1629/mm^2^, and 225 and 521/mm^2^ in the regressive and progressive lesion, respectively), and this time in line with the Danaher and MCP counter immune cell subsets count (Suppl. Figs. 2 and 3). PD-L1 expression was similar between the regressive versus progressive lesion in both patients, but was high in one while low in the other patient (Table 2).

For the latter group of patients with progressive lesions at a different site (patients 4–6), data are incomplete. In general, again a higher CD8 infiltration and higher IFNγ signature levels in the progressive lesion was observed (Table 2), while the Danaher and MCP counter immune cell subsets were inconclusive (Suppl. Figures 4 and 5).

**Fig. 2:**
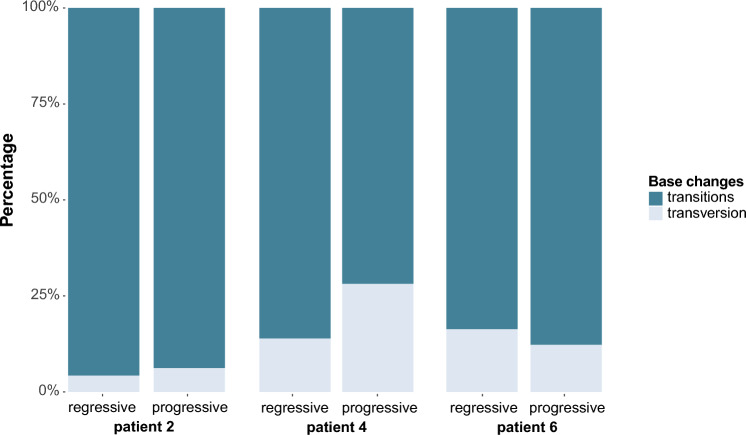
Overview of mutational profiles. Overview of transitions versus transversions of patients 2, 4 and 6

Due to lack of sufficient material, DNA sequencing analyses were only available for patient 2, 4 and 6. Patient 2 with a novel tumor lesion had only 2% difference in composition of mutational profile between lesions (Fig. 2), while TMB level (the total number of non-synonymous somatic mutations) was lower (-35%) in the novel lesion (Table 2). Patients 4 and 6, both with progressive lesions at a different site, had slight differences (14 and 4%, respectively) (Fig. 1), while TMB levels were evidently higher in the novel lesions (+ 540% and + 419%, respectively) (Table 2).

In addition, focused DNA mutation analyses were performed in both lesions (Suppl. Table 1). Only in patient 2 a pathogenic mutation (according to FATHMM prediction) was found in the regressive tumor lesion in the ATM gene (c.8494C > T) (Table 3). Although the progressive lesion of patient 4 had many mutations in the genes used for the focused DNA analyses, including different mutations in the HLA-A and HLA-C genes, the majority of these mutations was considered neutral according to FATHMM prediction (Table 3). No mutations were found in both lesions of patient 6 through focused DNA mutation analyses.

**Table 3:** Overview of focused DNA mutation analyses of samples

Patient	Lesion	Gene	Mutation		Mutation type	Variant	FATHMM prediction
2	Regressive	ATM	c.8494C > T	p.Arg2832Cys	Missense mutation	SNP	Pathogenic (0.96)
4	Regressive	APC2	c.3412G > A	p.Gly1138Arg	Missense mutation	SNP	–
4	Regressive	HLA-B	c.410A > C	p.His137Pro	Missense mutation	SNP	–
4	Regressive	APC2	c.3412G > A	p.Gly1138Arg	Missense mutation	SNP	–
4	Progressive	EARP1	c.35C > T	p.Thr12Ile	Missense mutation	SNP	Neutral (0.07)
4	Progressive	EGFR	c.1562G > A	p.Arg521Lys	Missense mutation	SNP	Neutral (0.05)
4	Progressive	FANCA	c.2426G > A	p.Gly809Asp	Missense mutation	SNP	Neutral (0.02)
4	Progressive	FANCA	c.796A > G	p.Thr266Ala	Missense mutation	SNP	–
4	Progressive	HLA-A	c.899_900inv	p.Leu300Pro	Missense mutation	DNP	–
4	Progressive	HLA-A	c.916A > G	p.Ile306Val	Missense mutation	SNP	Neutral (0.03)
4	Progressive	HLA-A	c.1005G > C	p.Lys335Asn	Missense mutation	SNP	Neutral (0.08)
4	Progressive	HLA-C	c.312C > A	p.Asn104Lys	Missense mutation	SNP	Neutral (0.01)
4	Progressive	HLA-C	c.302G > A	p.Ser101Asn	Missense mutation	SNP	Neutral (0.00)
4	Progressive	HLA-C	c.218C > A	p.Ala73Glu	Missense mutation	SNP	Neutral (0.04)
4	Progressive	NLRC5	c.1358C > T	p.Pro453Leu	Missense mutation	SNP	Neutral (0.03)
4	Progressive	NLRC5	c.1498 T > C	p.Cys500Arg	Missense mutation	SNP	Neutral (0.09)
4	Progressive	RFX5	c.1226C > G	p.Pro409Arg	Missense mutation	SNP	–
4	Progressive	TAPBP	c.779C > G	p.Thr260Arg	Missense mutation	SNP	Neutral (0.12)
4	Progressive	TNFRSF1A	c.362G > A	p.Arg121Gln	Missense mutation	SNP	–
7	Progressive	ERAP2	c.22G > A	p.Val8Ile	Missense mutation	SNP	–
7	Progressive	NLRC5	c.1934C > T	p.Pro645Leu	Missense mutation	SNP	–
7	Progressive	POLE	c.4514C > T	p.Pro1505Le	Missense mutation	SNP	Pathogenic (1.00)
8	Progressive	B2M	c.240G > A	p.Trp80Ter	Non-sense mutation	SNP	–
8	Progressive	PTEN	c.821G > A	p.Trp274Ter	Non-sense mutation	SNP	Pathogenic (1.00)
10	Progressive	ATM	c.8801C > T	p.Thr2934Ile	Missense mutation	SNP	–
10	Progressive	BTLA	c.157C > T	p.Pro53Ser	Missense mutation	SNP	–
10	Progressive	LAG3	c.484C > T	p.Leu162Phe	Missense mutation	SNP	–
10	Progressive	PD-1	c.782C > T	p.Ser261Phe	Missense mutation	SNP	–

*SNP* single nucleotide polymorphism, *DNP* double nucleotide polymorphisms

### Characteristics of acquired resistant lesions

Additional analyses were available for four patients with progressive tumor lesions after initial response (the responding tumor lesions of these patients were not available for analysis). High CD8 infiltration (range 762–1161 CD8^+^ cells/mm^2^) and relatively high IFN*γ* signature levels (range −0.177 to 1.108) were observed compared to both the regressive and progressive lesions of the previously described patients (Table 2).

DNA sequencing analyses were available of three patients. TMB levels were variable between these patients (range 327–1221) (Table 2). Two non-sense (stop gained) mutations in the B2M (c.240G > A) and PTEN (c.821G > A, pathogenic according to FATHMM prediction) genes of patients 8 were observed (Table 3). Patient 7 had one pathologic missense mutations in POLE (c.4514C > T), while patient 10 had only missense mutations. In the lesions of patient 9, no mutations were found through the focused analyses.

## Discussion

As the biological mechanisms underlying dissociated response to ICI are still largely unknown, we have investigated paired tumor lesions of patients with such a response. Tumor immune exclusion [27–29] was in general not observed, as in all progressive lesions immune cell infiltrates were found and in the majority even at higher levels than in the regressive tumor lesion. Due to the unavailability of viable tumor material, unfortunately no co-culture experiments dissecting tumor-specific T cell infiltrates from bystander T cell tumor infiltration could be performed. Thus, we could not confirm the notion that an immune exclusive phenotype [27–29] is responsible for the lack of response to ICI.

In addition, the genetic make-up of progressive tumor lesions did not deliver conclusive explanations for ICI resistance. In the three different groups of patients with paired tumor lesions, mutational profiles showed only 2–14% difference between regressive and progressive lesions. Progressive lesions at a different site, however, had a 429–540% increase in TMB levels, while only the newly emerged tumor lesion had a lower TMB level compared to their regressive lesions (−35%). A high TMB level has been associated with response to ICI in melanoma [30, 31], but no data are available on TMB levels within one patient in both regressive and progressive tumor lesions.

Furthermore, when analyzing for potential mutations responsible for tumor immune escape, only in one of the patients with a non-paired acquired resistant tumor lesion, stop-gaining mutations were found in the B2M and PTEN genes. B2M plays a role in stabilization of the MHC class I molecules at the cell surface, which on their turn are of importance in antigen presentation and the subsequent recognition of the immune system [32]. Alterations in this gene are previously described in melanoma patients with acquired resistance to ICI [28, 33] and also in patients with lung cancer [34] and mismatch repair-deficient cancers [35]. In our patient, we have not confirmed the loss of expression of B2M or MHC-I expression in the tumor by immunohistochemistry due to lack of sufficient tumor material. Data in pathways related to MHC expression (data not shown) were not easily to interpret as these levels are relative compared to the total patient set and no paired sample with known response at lesion-level was available for this patient.

PTEN loss has been previously described in one patient with melanoma who developed acquired resistance to ICI [36]. Loss of function of PTEN diminishes T cell priming; by loss of activation of phosphoinositide-3 kinase lipidation of autophagosome protein LC3 is inhibited and subsequently autophagy of tumor cells is inhibited [37]. PTEN negative melanoma tumors, defined by immunohistochemistry staining, are related to poor patient outcome and absence of T cell infiltration [38].

The other pathogenic mutation found in our analysis was in the POLE gene of another patient with a non-paired acquired resistant tumor lesion. DNA polymerase epsilon (POLE) plays, together with polymerase delta 1 (POLD1), an important role in DNA damage response and both have an important role in DNA replication by proofreading [39]. In a retrospective analysis of patients with various cancer types, pathogenic mutations in POLE were associated with clinical benefit to ICI [40]. Another study demonstrated that both POLE and POLD1 mutations could be a promising predictive biomarker of ICI response [41].

Other mechanisms of immune escape previously described in melanoma patients, such as mutations in the JAK1 and JAK2 gene [33, 42, 43], were not observed in our cohort.

All patients included in this work had favorable patient characteristics, with low lactate dehydrogenase (LDH) levels and a good performance status. These are the patients who often have durable benefit of ICI treatment [44, 45]. Our idea was that inclusion of patients with proved anti-tumor immune response and performing an intrapatient comparison with a progressive tumor lesion might be the cleanest approach to identify mechanism of ICI resistance, but this unfortunately failed in our hands.

Previous studies have shown low TMB levels, HLA loss, low CD8 infiltration, low LAG-3 or low TIM-3 expression to be associated with lower chance of response [12, 40, 43, 46], but when addressing our intrapatient analyses of regressive versus progressive tumor lesions, these parameters did not validate. Of interest, the majority of patients with higher levels of CD8 T cell infiltration and IFNγ expression in the progressive tumor lesions, did have response to the ICI line given after the biopsy was taken. Albeit our translational data of one metastatic lesion will not necessarily be a correct representation of response on patient-level, this is an interesting observation.

Although this descriptive study consists of a small number of patients, it provides nonetheless an overview of (largely) paired tumor lesion analyses in this field in which a lot is still to be discovered and understood. Of note, due to the normalization methods applied to RNA sequencing data, levels described are relatively high or low within this specific subset of patients, which could make comparison to data in other studies difficult. A challenge arising with our DNA sequencing data is that one comes across different mutations of which the consequences are not known. The FATHMM prediction tool [19] helps herein by providing a predictive neutral or pathogenic score based on prior literature, but nonetheless the largest part of mutations found in our patients could not be traced back to previous studies.

As in the majority of patients the paired tumor lesions did not origin from the same organ, organ-specific effects could affect our results [47]. A study that looked into site-specific patterns of response to ipilimumab and nivolumab demonstrated a significant inter- and intrapatient heterogeneity in response and progression [48]. This presumably reflects differences in underlying molecular heterogeneity, as, for example, the liver has an immune suppressive microenvironment and is associated with worse outcome than metastases in the lung [48, 49]. In the liver sample of patient 3, a low IFNγ score was seen, which can probably not fully be contributed to the tumor itself but can partly be explained by the liver microenvironment. The different sites of the tumor lesions and the difference in immunogenicity depending on the organ in which the metastasis developed [47, 50] complicate the comparison of our data between regressive and progressive tumor lesions.

Our study confirms the challenges in identifying mechanisms of immune escape, which seem to be very individual per patient, and even per metastasis, while parameters associated with response (such as high neoantigen load, CD8 infiltration and high IFN*γ* signature) are easier to identify. Hence, we still have a long way to go until we understand resistance mechanisms to ICI.

### Supplementary Information

Below is the link to the electronic supplementary material.

Supplementary file1 (PDF 705 kb)

## Data Availability

Data are available upon reasonable request for academic use and within the limitations of the provided informed consent. Every request will be reviewed by the institutional review board of the NKI; the researcher will need to sign a data access agreement with the NKI after approval.
