# Supplementary material for: Tumor characteristics of dissociated response to immune checkpoint inhibition in advanced melanoma
Source: Cancer Immunol Immunother. 2024 Jan 27;73(2):28. doi: 10.1007/s00262-023-03581-6 (PMC10821835; doi:10.1007/s00262-023-03581-6)
Supplement: Supplementary file 1 — Supplementary file1 (PDF 705 kb) [file 262_2023_3581_MOESM1_ESM.pdf]

SUPPLEMENTARY APPENDIX

Supplement to: 'Tumor characteristics of dissociated response to immune checkpoint inhibition in advanced melanoma'

Contents

Suppl. Table 1. Treatment details per patient 2

Suppl. Table 2. Overview of DNA mutation in focused analyses 3

Suppl. Figure 1. MCP counter and Danaher gene expression data of patient 1 4

Suppl. Figure 2. MCP counter and Danaher gene expression data of patient 2 5

Suppl. Figure 3. MCP counter and Danaher gene expression data of patient 3 6

Suppl. Figure 4. MCP counter and Danaher gene expression data of patient 4 7

Suppl. Figure 5. MCP counter and Danaher gene expression data of patient 5 8

Suppl. Table 1. Treatment details per patient

Patient	prior DTIC	BOR to DITC	prior ICI	BOR to prior ICI	ICI treatment	BOR to ICI	subsequent treatment	BOR to subsequent treatment
pat 1					IPI+NIVO	PD*		
pat 2					PEM	PR	PEM	PD
pat 3	yes	SD	IPI	PD	PEM	PR		
pat 4					NIVO	PR		
pat 5			IPI + PEM	NE + PR	PEM	PR		
pat 6					NIVO	MR	NIVO	
pat 7					PEM	PD	IPI	PR
pat 8					IPI+NIVO	PR	NIVO	
pat 9					NIVO	PR		
pat 10					PEM	CR		

*response on subsequent radiotherapy treatment.
BOR: best overall response; ICI: immune checkpoint inhibition; IPI: ipilimumab; NIVO: nivolumab; PEM: pembrolizumab; PD: progressive disease; PR: partial response; MR: mixed response; CR: complete response; NE: non-evaluable.

Suppl. Table 2. Overview of DNA mutation in focused analyses

Antigen processing + presentation	Immune checkpoints	Resistance mechanisms	DNA damage response
TAP1	CTLA-4	PTEN	BRCA1
TAP2	PD-1	EGFR	BRCA2
TAPBP	LAG-3	MYC	ATM
TAPBPL	TIM-3		POLE
HLA-A	TIGIT	**β-catenin signaling**	FANCE
HLA-B	VISTA	SOX2	ERCC2
HLA-C	BTLA	SOX11	MDH6
B2M		WNT7B	
CALR	**Interferon + JAK/STAT**	EFNB3	**TNF signaling**
NLCR5	IFNG	APC2	CASP8
PSMB9	IRF	TCF1	TNFRS1A
PSMB10	STAT1	cMYC	ADO
PSME1	JAK1	TCF12	AQSTM1
PSME2	JAK2	VEGFA	GOSR1
PSME3	IFNGR1		
RFX5	IFNGR2	**Chemokines**	
HSP90AB1	SOCS1	CXCL9	
CANX	PIAS4	CXCL10	
ERp57		CCR4	
ERAP1		CCR7	
ERAP2			

Suppl. Figure 1. MCP counter and Danaher gene expression data of patient 1

A

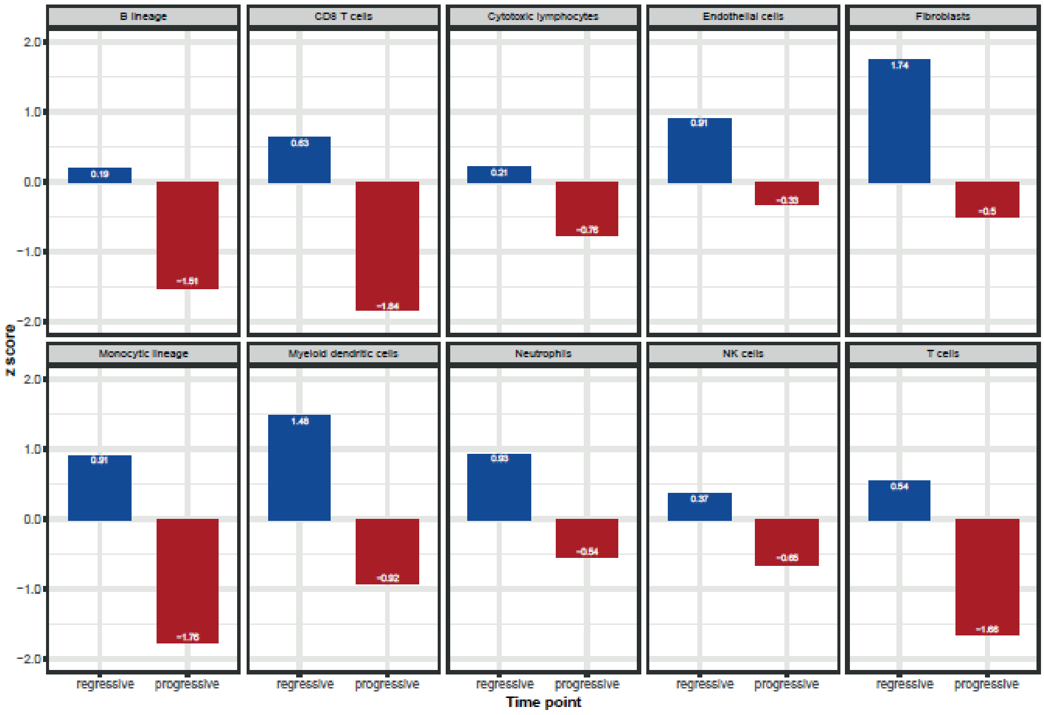

B

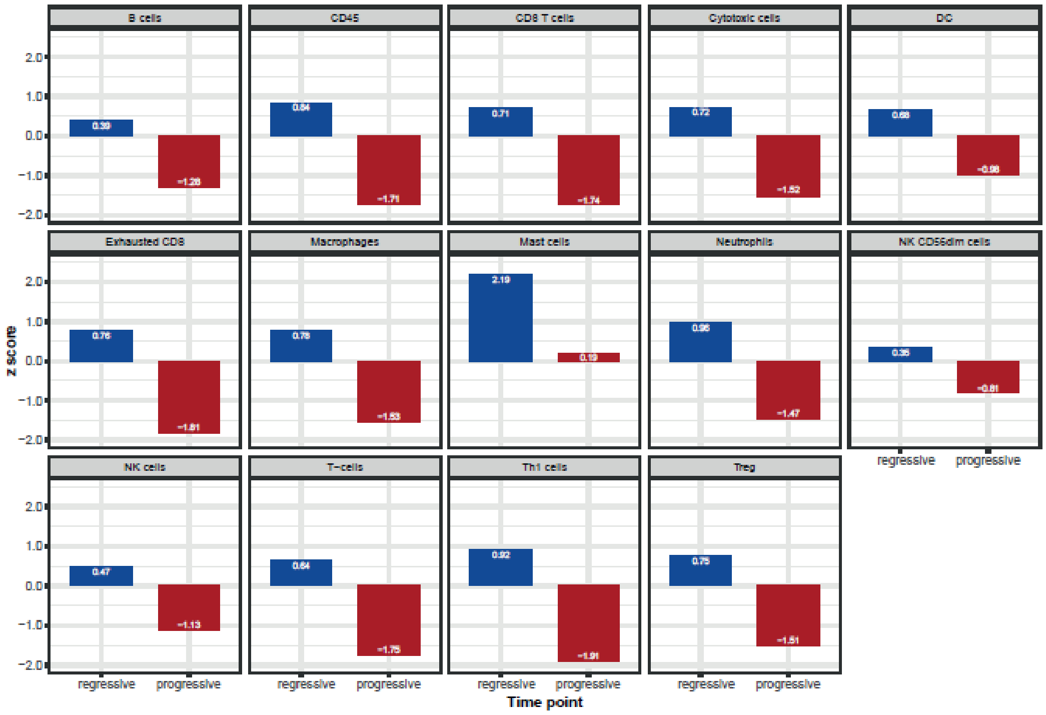

A. Overview of expression of MCP counter genes, in blue for regressive and in red for progressive tumor lesion.
B. Overview of expression of Danaher genes, in blue for regressive and in red for progressive tumor lesion.

Suppl. Figure 2. MCP counter and Danaher gene expression data of patient 2

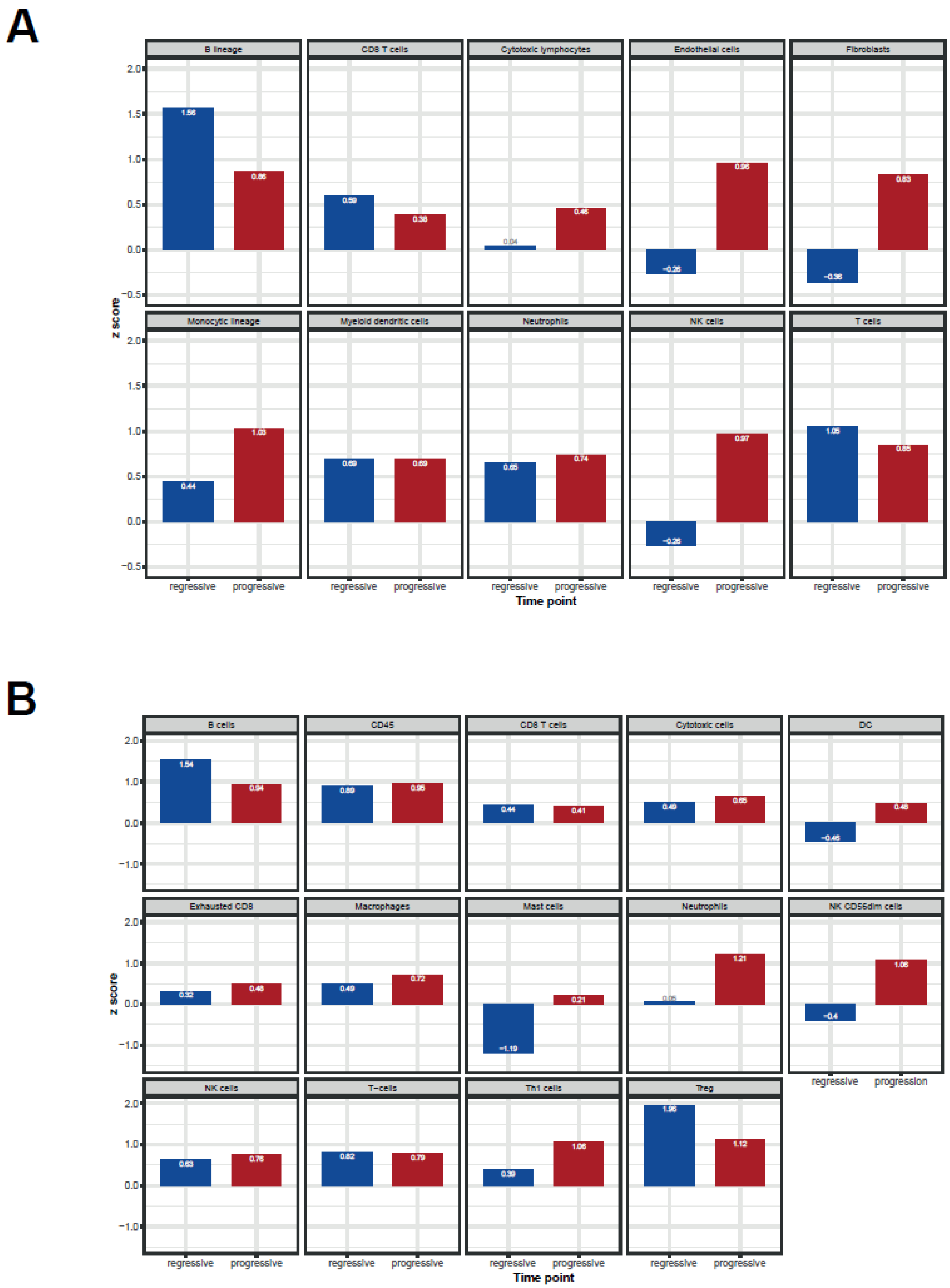

A. Overview of expression of MCP counter genes, in blue for regressive and in red for new progressive tumor lesion.
B. Overview of expression of Danaher genes, in blue for regressive and in red for new progressive tumor lesion.

Suppl. Figure 3. MCP counter and Danaher gene expression data of patient 3

A

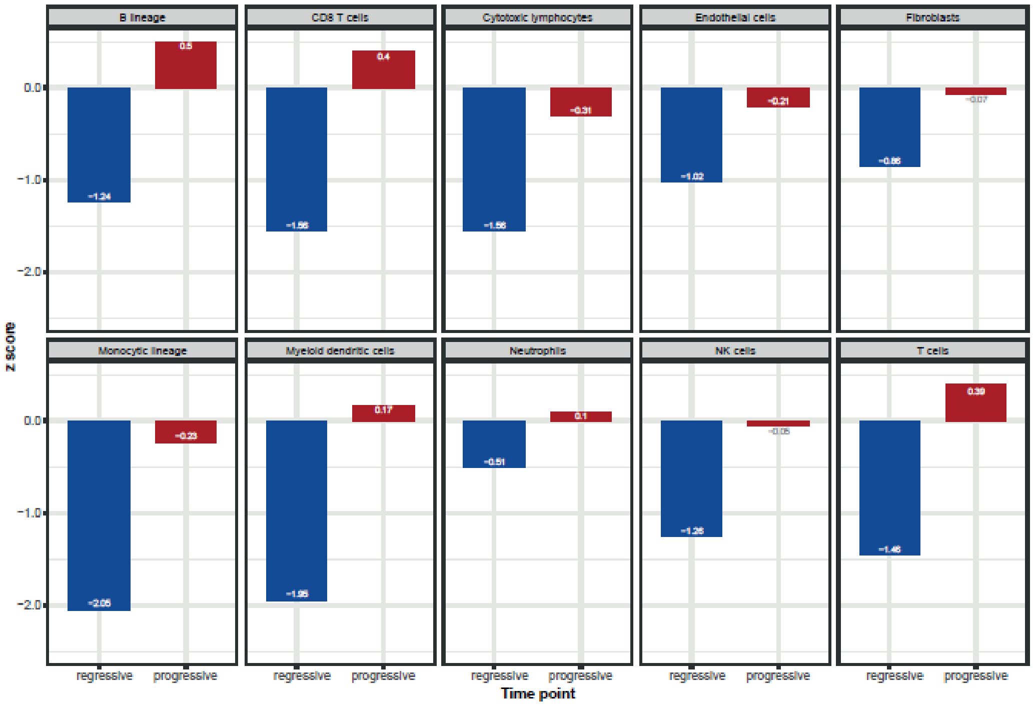

B

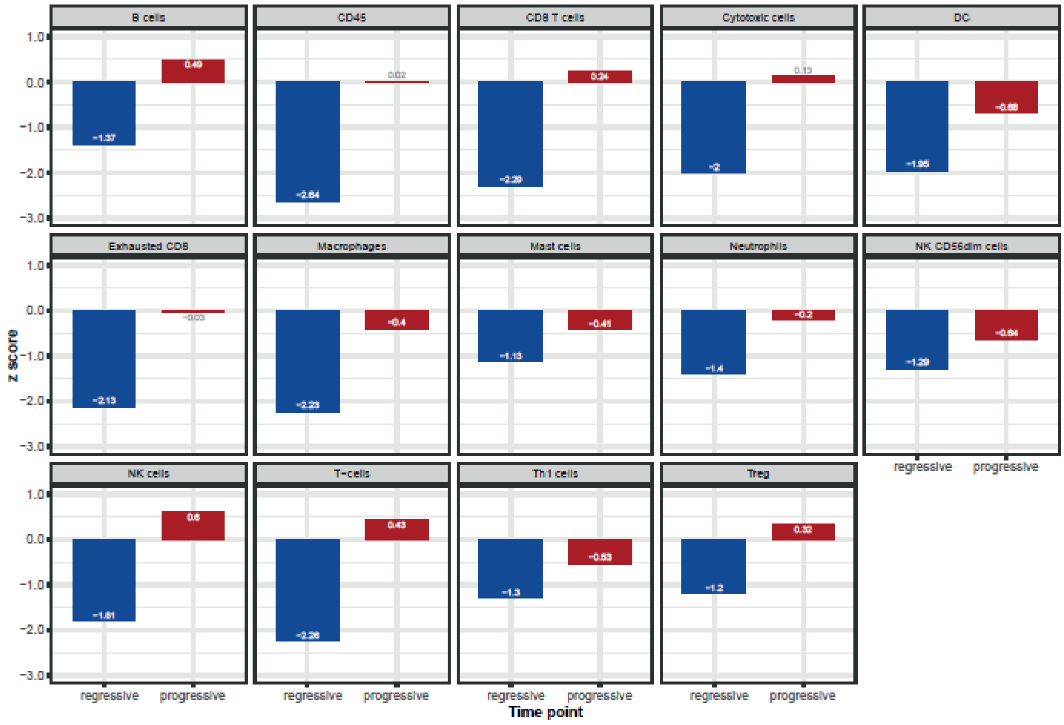

A. Overview of expression of MCP counter genes, in blue for regressive and in red for new progressive tumor lesion.

B. Overview of expression of Danaher genes, in blue for regressive and in red for new progressive tumor lesion.

Suppl. Figure 4. MCP counter and Danaher gene expression data of patient 4

A

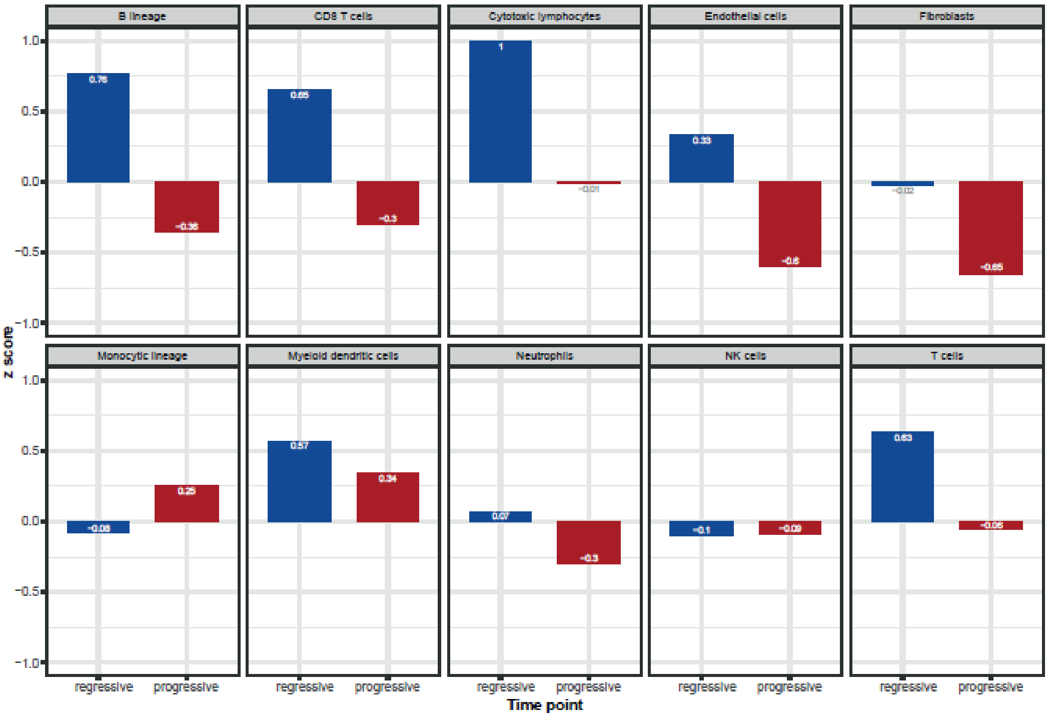

B

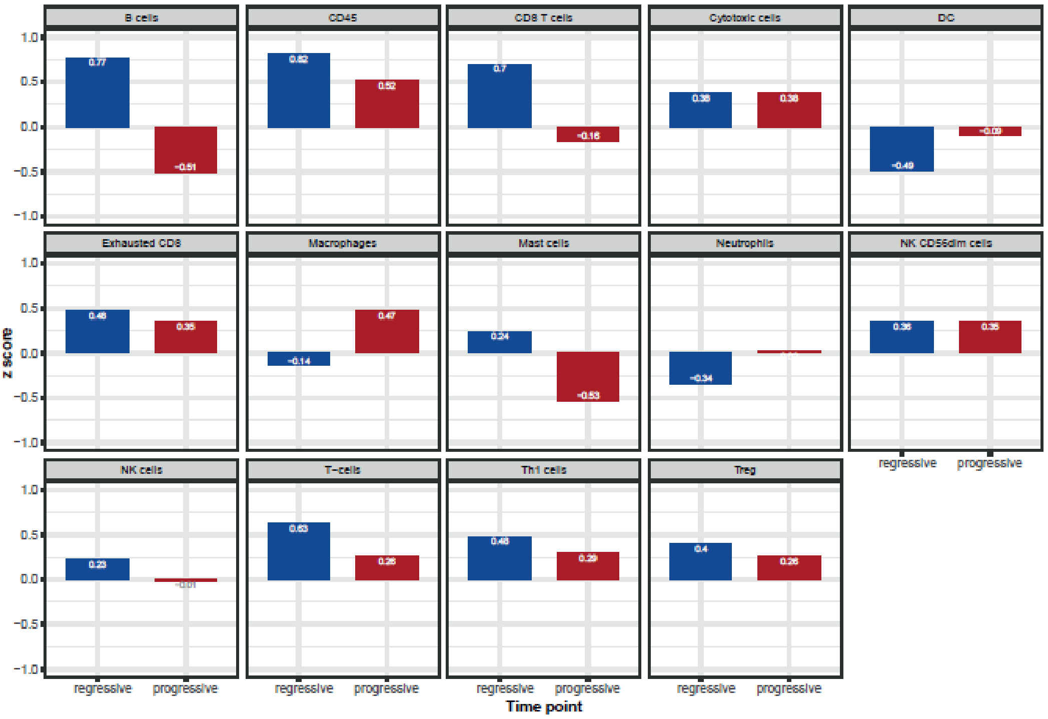

A. Overview of expression of MCP counter genes, in blue for regressive and in red for progressive tumor lesion.
B. Overview of expression of Danaher genes, in blue for regressive and in red for progressive tumor lesion.

Suppl. Figure 5. MCP counter and Danaher gene expression data of patient 5

A

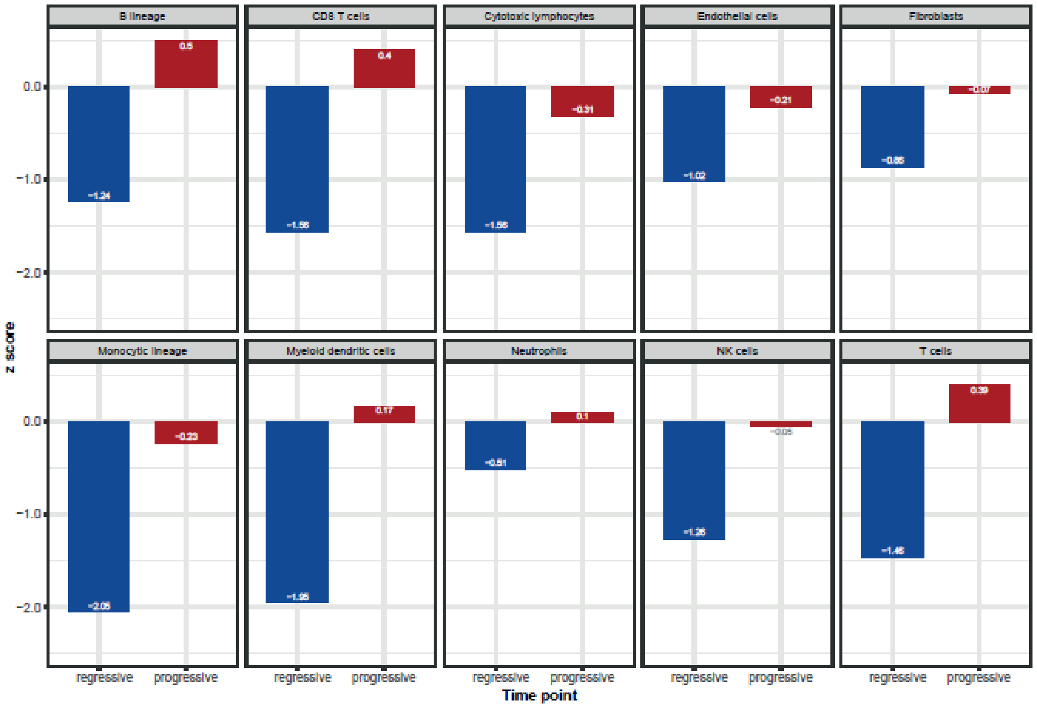

B

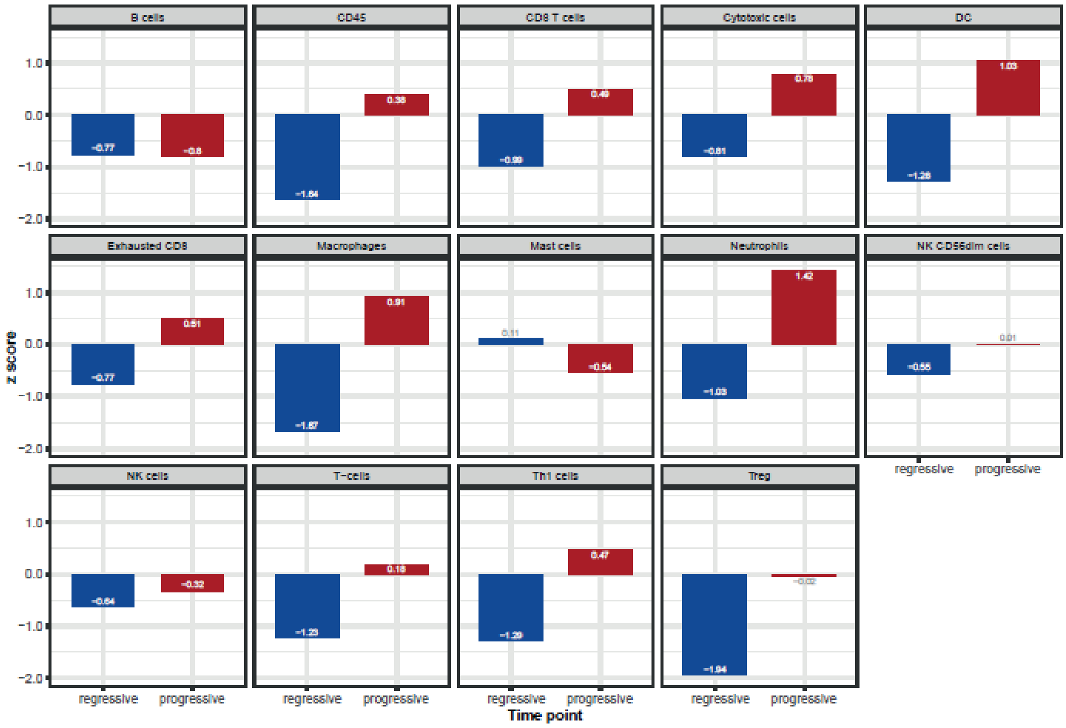

A. Overview of expression of MCP counter genes, in blue for regressive and in red for progressive tumor lesion.

B. Overview of expression of Danaher genes, in blue for regressive and in red for progressive tumor lesion.
